# Revisiting batch effect correction: a structured problem formulation and novel solutions

**DOI:** 10.1093/bioinformatics/btag735

**Published:** 2026-10-15

**Authors:** Avraham Harrar, Uri Shaham

**Affiliations:** Department of Computer Science, Bar Ilan University, Ramat Gan 5290002, Israel; Department of Computer Science, Bar Ilan University, Ramat Gan 5290002, Israel

## Abstract

**Motivation:**

Existing methods for batch effect removal often suffer from an ill-defined problem formulation and rely on unverified assumptions, limiting their robustness and generalizability. This study proposes a principled approach to systematically address these limitations.

**Results:**

We present two novel methodologies for removing systematic batch effects: an unsupervised approach based on independent component analysis and a supervised approach using a domain adaptation technique. Both methods are grounded in rigorous mathematical principles. Our approaches outperform existing methods across multiple datasets.

**Availability and implementation:**

The implementation of our methods, including all code, datasets, experimental workflows, and supplementary material, is available in Python and has been made publicly accessible on GitHub: https://github.com/shaham-lab/revisiting-batch-effect-removal.

## 1 Introduction

In recent years, advancements in high-throughput technologies have significantly enhanced our capacity to investigate complex biological systems. A critical challenge in this domain is the integration of data from multiple measurement sources to improve biological insights. One particular challenge arises when aggregating data from different experimental batches, even when utilizing the same sequencing technology. This often introduces a batch effect, a form of systematic noise induced by non-biological factors that are difficult to model.

For instance, two distributions of high-dimensional molecular data derived from identical blood samples (technical replicates) analyzed in separate experimental batches may exhibit substantial variation due to differences in batch-specific conditions. These batch effects introduce systematic biases, potentially leading to misleading statistical inferences and obscuring the true biological signal. The underlying causes of batch effects are often complex and influenced by numerous unknown factors, making it difficult to precisely model their impact on observed data.

While numerous batch correction algorithms have been developed, the problem itself remains ill-defined (Yu *et al.* 2023). A more rigorous characterization of the problem and its underlying assumptions is essential. Specifically, defining a data generation model and explicitly characterizing the manifestation of batch effects could provide a theoretical foundation for designing more effective correction methods. This study seeks to address these issues by proposing a principled approach to batch effect correction. Several key challenges in existing methodologies motivate our research:

1. Many batch correction techniques rely on principal component analysis [PCA (Jolliffe 2002)] for dimensionality reduction. However, PCA is a linear transformation and may fail to capture the complex, non-linear relationships that often characterize batch effects in biological data. This limitation can lead to incomplete correction, leaving residual non-linear artifacts in the data.
2. A prevalent issue in contemporary bioinformatics is that many batch effect correction methods operate exclusively within a shared latent space. This approach often compromises the interpretability of the results in the context of the original data representation, and limits the ability to synthesize corrected data back into the input space.
3. The theoretical assumptions required to guarantee the identifiability of the true biological signal amidst batch-induced variation remain an open question, leaving many methods without a formal basis for their correctness.

To address these challenges, we introduce two novel methodologies. The first is an unsupervised approach designed to mitigate non-linear batch effects, which can be conceptualized as a special case of independent component analysis (ICA) (Hyvärinen *et al.* 2001). This method provides a robust framework for reducing batch-induced distortions in data analysis. The second is a supervised technique that generalizes mutual nearest neighbor (MNN)-based approaches. This method facilitates improved cell-type classification and enables the construction of a robust representation that effectively aligns batch effects using labeled data.

### 1.1 Related works

The literature on batch effect removal is vast and cannot be reviewed in full detail within the scope of this article. However, we identify three major categories of methods in the field, which are discussed below.

The first category includes methods such as MNNs, fastMNN (Haghverdi *et al.* 2018), Scanorama (Hie *et al.* 2019), and BBKNN (Polanski *et al.* 2020). These techniques focus on identifying MNNs—pairs of cells from different batches that are closest to each other in terms of gene expression profiles.

The second category encompasses methods such as Seurat (Stuart *et al.* 2019), Harmony (Korsunsky *et al.* 2019), and LIGER (Liu *et al.* 2020), which project data into a shared latent space using linear techniques such as PCA or matrix factorization. While differing in methodology, both categories share common limitations. They often rely on linear dimensionality reduction techniques to manage the computational complexity of large datasets. However, such approaches may fail to capture non-linear batch effects, leading to incomplete correction. Moreover, the reduction in dimensionality can result in loss of important biological information and compromise interpretability—either by discarding variation relevant to the signal or by transforming the data into a latent space that obscures the contribution of original features.

The third category includes deep learning-based methods, which leverage the scalability and representational power of neural networks to address batch effects. For example, Shaham *et al.* (2017) employed residual neural networks to minimize the maximum mean discrepancy (MMD) between distributions, while Lotfollahi *et al.* (2019) introduced scGen, a variational autoencoder (AE) framework for modeling perturbation responses and correcting batch effects. SAUCIE (Amodio *et al.* 2019) uses a sparse AE architecture to jointly perform batch correction, clustering, and imputation, encouraging biologically meaningful representations through a sparsity constraint. DeepST (Dong *et al.* 2022) integrates graph neural networks with adversarial training, using gradient reversal layers to enforce batch-invariant embeddings. Similarly, deepMNN (Li *et al.* 2023) incorporates MNN information into a deep neural network that balances between preserving biological variance and minimizing batch-induced differences. While these methods have demonstrated strong empirical performance, a common limitation is the lack of theoretical guarantees for complete separation of biological signals from batch effects. Our methods belong to this family of deep learning-based approaches. However, they directly address this critical gap by proposing architectures grounded in fundamental theoretical principles, offering theoretical guarantees for removing batch effects.

### 1.2 Learning setup and problem formulation

It is reasonable to assume that biological measurements are generated as a function of two independent random variables, one that corresponds to the inherent biological state and is independent of the measuring procedure and one corresponding to the measurement itself and is independent of the biology. To formalize this, we consider the following mathematical setup: let X∈X be an arbitrary random variable corresponding to a data measurement. We assume that *X* is generated as X=f(S,T), where *f* is an invertible function, S∈S is a random variable describing the biological state, and T∈T={1,…,B} identifies a measurement condition, indicating a “batch”. We assume that *S* and *T* are independent.

Let P be a distribution on X×T, whose density is *P*. Let $P^{t}$ be the distribution induced from P on X, given T=t, with density $P^{t}$, that is $P^{t}(X=x)=P(X=x|T=t)$. We refer to a *batch* as a sample $B^{t}=\{x_{1},\ldots ,x_{n_{t}}\}$ from $P^{t}$.

We distinguish between supervised and unsupervised setups, as follows.

- In the unsupervised regime, we view *X* as a biological measurement.
- In the supervised regime, we consider *X* to be a labeled example X=(Z,Y), where Z∈Z is the measurement and Y∈Y is its label, e.g. cell type.

The following definitions apply to the supervised setup.

Definition 1 We denote by $P_{z}^{t}$ the conditional distribution on Y, obtained from $P^{t}$ given Z=z, and the corresponding density $P_{z}^{t}(Y=y):=P^{t}(Y=y|Z=z)$.

Definition 2 Let $Q^{t}$ denote the marginal distribution over Z equipped with the density function

$$Q^{t}(z)=\int_{y\in Y}P^{t}(z,y)dy,$$

for any z∈Z.

Remark 1 Consider a map ϕ from the input space Z to some embedding space E. The embedding space E can be viewed as an analog or representation of the original input space Z.

Definition 3 Let $\tilde{P^{t}}$ be the joint distribution defined on the embedding-output space that is induced by the marginal pushforward distribution, i.e. the joint distribution of (ϕ(z),y), where $(z,y)∼P^{t}$.

Definition 4 (Hypothesis and error). A hypothesis is a function h:Z→Y. The generalization error of a hypothesis *h* concerning a batch *t* is $\xi_{P^{t}}(h)=E_{(z,y)∼P^{t}}\ell (h(z),y)$, where $\ell :Y\times Y\to R^{+}$ is a loss function, typically determined by the specific downstream task, such as the 0–1 loss or the $\ell_{1}$ loss.

Definition 5 (Total variation distance). Consider a measurable space (Ω,F) and probability measures $Q^{t}$ and $Q^{t^{\prime }}$ defined on (Ω,F). The total variation distance between $Q^{t}$ and $Q^{t^{\prime }}$ is defined as

$$d(Q^{t},Q^{t^{\prime }})=\underset{A\in F}{\text{sup}}\left|Q^{t}(A)-Q^{t^{\prime }}(A)\right|,$$

where $Q^{t}(A)=\int_{z\in A}Q^{t}(z)dz$, This is the largest absolute difference between the probabilities that the two probability distributions assign to the same event.

Definition 6 (Cross-batch conditionals agreement (CBCA) error) We define the cross-batch conditionals agreement (CBCA) error between batches $t,t^{\prime }$ as

(1) $$\begin{array}{c} \chi_{t,t^{\prime }}\equiv E_{z∼Q^{t}}E_{\begin{array}{c} y∼P_{z}^{t},\\ y^{\prime }∼P_{z}^{t^{\prime }} \end{array}}\ell (y,y^{\prime }). \end{array}$$

CBCA error quantifies the inconsistency between conditional label distributions across batches, conditioned on the same input samples. For two batches *t* and $t^{\prime }$, the CBCA error $\chi_{t,t^{\prime }}$ is defined as the expected loss between label samples $y∼P_{z}^{t}$ and $y^{\prime }∼P_{z}^{t^{\prime }}$, where $z∼Q^{t}$ are samples drawn from batch *t*. This metric captures the extent to which conditional labels vary across batches for the same inputs and serves as a measure of cross-batch agreement.

#### 1.2.1 Unsupervised batch effect removal

Let ${\left\{(x_{i},t_{i})\right\}}_{i=1}^{n}$, be *n* realizations from P such that for all i=1,…,n, $x_{i}=f(s_{i},t_{i})$, for some unobserved $s_{1},\ldots ,s_{n}$. We state two goals.

- Analysis: we aim to recover the realizations $s_{1},.\ldots s_{n}$ (the biological states). This corresponds to batch effect removal, via learning an encoding that represents merely biological conditions and lacks variability resulting from batch effects.
- Synthesis: for any (new) realization $t^{\prime }$ of *T* (the batch component), we aim to convert an instance $x=f(s_{i},t)$ for any i=1,…,n, to $x^{\prime }=f(s_{i},t^{\prime })$. This corresponds to calibration of different batches in input space.

#### 1.2.2 Supervised batch effect removal

For a two-batch case, assume T={0,1}. We assume the availability of two datasets, one from each batch, $D^{0}={\left\{(z_{i},y_{i})\right\}}_{i=1}^{N_{0}}$ and $D^{1}={\left\{(z_{i},y_{i})\right\}}_{i=1}^{N_{1}}$, containing iid samples drawn from $P^{0}$ and $P^{1}$, respectively. We refer to $D^{0}$ as the source sample and to $D^{1}$ as the target sample. We also assume that $N_{0}$ is significantly larger than $N_{1}$.

We state two goals:

- Low generalization error on future source and target samples: given some loss objective term $\ell :Y\times Y\to R^{+}$, we aim to learn a hypothesis h:Z→Y which achieves low generalization error on source and target samples: $\xi^{0}=E_{(z,y)∼P^{0}}\ell (y,h(z))$, $\xi^{1}=E_{(z,y)∼P^{1}}\ell (y,h(z))$.
- Batch-invariant representation: given a batch label t∈T and a sample $z\in D^{t}$, the goal is to learn a representation ϕ(z) that is statistically independent of the batch component *T*, i.e. ϕ(z)⊥⊥T.

Remark 2
$N_{0}\gg N_{1}$ :

This assumption of imbalance, where the source batch size $N_{0}$ is significantly larger than the target batch size $N_{1}$, introduces a substantial challenge. In scenarios where both source and target batches are sufficiently large, it is relatively straightforward to minimize generalization error by directly training on the target batch. In contrast, our method focus is on the more difficult regime where the target batch is small. This setting complicates the learning process.

### 1.3 The proposed methods

#### 1.3.1 Unsupervised batch effect removal

Here, we view the problem of batch effect removal as a special case of ICA (Hyvärinen *et al.* 2001). ICA considers a setup where an observed signal is generated as a mixture of statistically independent components. When the mixture is linear and the components are non-Gaussian, ICA has been shown to be identifiable (Hyvärinen *et al.* 2001). However, it is known that ICA is not identifiable in the general case. One of our recent papers (Shaham *et al.* 2022) presented an approach for provable recovery of a single independent component that naturally fits the scenario just presented. In particular, we considered data that is generated as a non-linear mixture of two statistically independent components, one of which is observed, and the goal is to recover the latent component.

In our scenario, as outlined in Section 3, *X* is generated by two random variables, *S* and *T*. *T* represents the batch, while *S* represents the unobserved biological state. For calibration purposes, it is imperative to recover *S*, which is precisely addressed in this article.

As illustrated in Fig. 1a, our approach utilizes an AE with a discriminator. The AE model is denoted by (ϕ,D), where the encoder ϕ maps inputs *x* to codes $s^{\prime }$ (analysis), and the decoder *D* maps code-condition tuples $\left(s^{\prime },t\right)$ to input reconstructions $\hat{x}$ (synthesis), i.e. $x\overset{\phi }{\to }s^{\prime }$ and $(s^{\prime },t)\overset{D}{\to }\hat{x}$. During inference, the encoder ϕ extracts the batch-invariant code $s^{\prime }$ from an input *x*, as shown in Fig. 1b. The decoder *D* then synthesizes the reconstruction ${\hat{x}}^{\prime }$ using $s^{\prime }$ and a target batch label $t^{\prime }$, as depicted in Fig. 1c.

**Figure 1:**
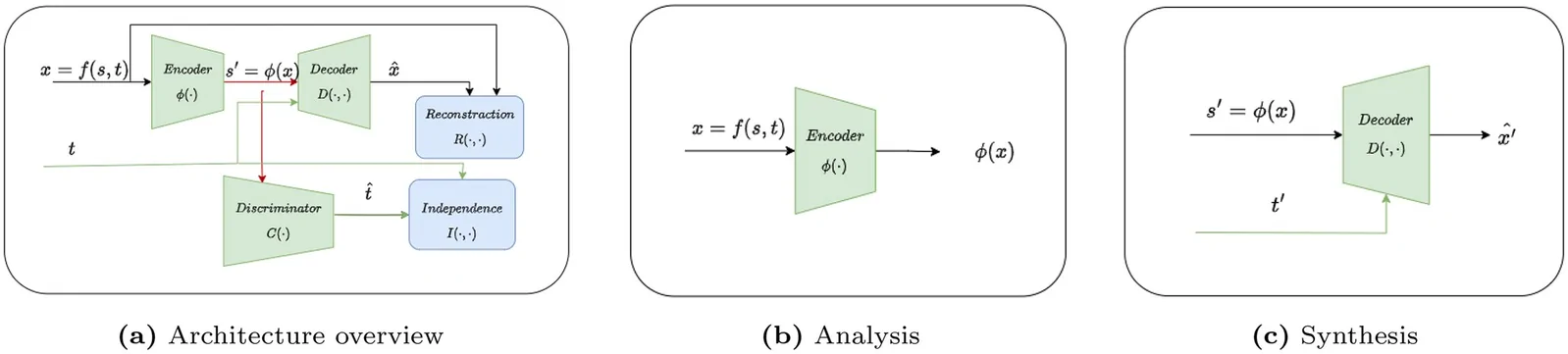
Architecture overview. The model is based on an autoencoder architecture comprising an encoder ϕ(·), a decoder D(·,·), and a discriminator C(·). An input x=f(s,t) is encoded into a latent representation $s^{\prime }=\phi (x)$, and the decoder reconstructs the sample as $\hat{x}=D(s^{\prime },t)$, aiming to recover the original input using the inferred biological signal $s^{\prime }$ and the batch label *t*. The reconstruction loss R(·,·) (e.g. $\ell_{1}$, $\ell_{2}$) quantifies the difference between x and $\hat{x}$. The discriminator predicts the batch label *t* from $s^{\prime }$, while the independence loss I(·,·) penalizes batch-specific information in $s^{\prime }$, encouraging batch-invariant representations. During inference, the encoder ϕ maps input *x* to $s^{\prime }=\phi (x)$ that is independent of the batch label *T*. The decoder *D* can then synthesize a new sample ${\hat{x}}^{\prime }=D(s^{\prime },t^{\prime })$ in the input space, enabling cross-batch translation.

To ground this architecture, the training objective must explicitly enforce the statistical independence of the biological state *S* from the batch *T*. The encoder ϕ maps the input *x* to a latent representation $s^{\prime }=\phi (x)$ that aims to preserve biological signal. The decoder *D* reconstructs the original input from $s^{\prime }$ and a batch label *t*.

To guarantee that this latent representation $s^{\prime }$ is entirely free of batch effects, we employ an adversarial discriminator C:E→T. In the multi-batch setting, *C* is formulated as a multi-class classifier that predicts the batch label directly from the latent representation. Specifically, it is trained to approximate the batch-label distribution by minimizing the cross-entropy loss *I* between the true batch label *t* and the predicted probabilities C(ϕ(x)):


(2) $$L_{\text{disc}}=\underset{C}{\min}I(t,C(\phi (x)))$$

By minimizing this loss, *C* establishes a global adversarial gradient. Conversely, the encoder and decoder are trained jointly to minimize the adversarial autoencoder loss, effectively fooling the discriminator while maintaining reconstruction fidelity. The underlying rationale for the discriminator design follows the Hirschfeld–Gebelein–Rényi (HGR) maximal correlation theorem, which characterizes the optimal dependence measure between representations under non-linear transformations, and motivates learning a discriminator that captures maximal statistical dependence between latent embeddings and batch labels


(3) $$L_{\text{AE}}=\underset{\phi ,D}{\min}\left[R(x,D(\phi (x),t))-\lambda I(t,C(\phi (x)))\right]$$

The reconstruction loss R(x,D(ϕ(x),t)) ensures that the latent representation retains enough information to accurately reconstruct the input when conditioned on a batch label. The second term penalizes the encoder for allowing batch-specific information to persist in the representation, as detected by the discriminator. The hyperparameter λ balances the tradeoff between reconstruction quality and batch invariance.

It was shown in Shaham *et al.* (2022) that if the encoding learned by the encoder is independent of the batch, and the reconstruction produced by the decoder is perfect, then the encoding $s^{\prime }=\phi (x)$ is an entropy-preserving representation of the biological component *S*. Under these conditions, the encoding captures the biological state and contains no information about the batch. For completeness, we state the result below and provide the full proof in the Supplementary Material, available as supplementary data at *Bioinformatics* online. Let H(S) denote the entropy of a random variable *S*.

Theorem 3 (Analysis) Suppose we train the autoencoder model to zero generalization loss, i.e. $\hat{X}=X$, and impose on the biological code that $S^{\prime }⊥⊥T$. Then $H(S)=H(S^{\prime })$.

This theorem supports **Analysis Goal 3.1** by establishing that the latent representation $S^{\prime }$ retains the full entropy of the biological signal *S* while being statistically independent of the batch label *T*. Consequently, the representation encodes only biologically relevant variation, free from batch-specific confounding, and is thus suitable for downstream analysis.

Theorem 4 (Synthesis) Let x=f(s,t) and $x^{\prime }=f(s,t^{\prime })$ be two samples with an identical biological state s but different batch labels t and $t^{\prime }$, respectively. Under the assumptions of Theorem 3, the model can synthesize sample $x^{\prime }$ using the information from x and the target batch identifier $t^{\prime }$. Formally:

$$D(\phi (x),t^{\prime })=x^{\prime }.$$

This theorem, whose proof is in the Supplementary Material, available as supplementary data at *Bioinformatics* online, supports our **Synthesis Goal 3.1**, demonstrating that the decoder can synthesize a sample from batch t into its corresponding realization in batch $t^{\prime }$. Thus, it enables batch effect removal directly in the **input space**, while preserving the underlying biological signal *s*.

##### 1.3.1.1 Theoretical bounds in the approximate regime

While the guarantees of Theorem 3 and the synthesis theorem establish our framework’s ideal behavior, they rely on perfect reconstruction ($R(X,\hat{X})=0$) and exact independence. To better reflect the realistic optimization dynamics of our adversarial setup, we relax these assumptions to consider approximate model convergence. Specifically, we adopt Assumption 6 (independence bound) and invoke Lemma 4 (conversion error bound) from Shaham *et al.* (2022). We denote by ϵ the uniform upper bound on the reconstruction error in the data space, i.e. $\|x-\hat{x}{\|}_{2}\leq \epsilon$, and by $\epsilon^{\prime }$ the independence error bound in the latent space. Under the assumption that the decoder D(·,t) is *L*-Lipschitz continuous with respect to its latent input, the conversion error $\epsilon_{\text{conv}}=\|x^{\prime }-{\hat{x}}^{\prime }{\|}_{2}^{2}$ satisfies:


$$\epsilon_{\text{conv}}\leq 2\left(L\epsilon^{\prime }+\epsilon^{2}\right).$$

This result implies that approximate independence in the latent space, coupled with controlled reconstruction error, guarantees bounded deviation during cross-batch synthesis. Crucially, it ensures that residual batch effects in the original space remain small and do not grow arbitrarily due to imperfect factorization. Full proofs and derivations are provided in the Supplementary Material (see page 4, Proof of Conversion Error Bound), available as supplementary data at *Bioinformatics* online.

#### 1.3.2 Latent space stratification and optimal transport coupling

In realistic settings, shared biological populations may coexist with batch-specific states or unequal cell-type proportions. To handle this, we first perform latent stratification and then apply optimal transport (OT) coupling only where independence constraints are appropriate. All cells from multiple batches are mapped into a shared latent space using an encoder $f_{\phi }$, producing a latent representation $Z=[Z^{\left(1\right)},\ldots ,Z^{\left(B\right)}]$, where *B* denotes the number of batches. We then apply adaptive *K* -means clustering (K∈[5,32]) on the full latent space. Clusters containing cells from more than one batch are defined as mutual clusters, while clusters containing cells from a single batch are defined as unique clusters. Formally, let $C_{\text{mut}}$ denote clusters shared across batches and $C_{\text{unq}}$ denote batch-specific clusters. This induces a partition of cell indices into mutual pools $P_{\text{mut}}^{\left(b\right)}$ and unique pools $P_{\text{unq}}^{\left(b\right)}$ for each batch b. Importantly, this stratification is independent of the coupling mechanism and is used only to determine where independence constraints should be applied. At each iteration, a Bernoulli variable δ∼Bernoulli(0.5) selects either mutual or unique pools for minibatch construction. Only mutual minibatches are used for independence enforcement. For mutual minibatches between a source batch 0 and target batch 1, we compute the latent cost matrix:


$$C_{ij}^{\text{lat}}=\|z_{0}^{\left(i\right)}-z_{1}^{\left(j\right)}{\|}_{1}$$

We then solve an entropically regularized OT problem (Sinkhorn) to obtain soft cross-batch couplings. These couplings are used exclusively in the batch-independence objective to align shared biological structure. Unique minibatches are excluded from the independence constraint, since enforcing alignment on batch-specific populations may remove meaningful biological variation. However, they remain fully included in reconstruction and generative objectives. This separation ensures that OT coupling is applied only where cross-batch correspondence is meaningful, while preserving batch-specific and rare populations.

#### 1.3.3 Supervised batch effect removal

While numerous batch effect correction methods exist, they often assume balanced sample sizes or unsupervised settings. These assumptions do not hold in many real-world scenarios—e.g. in rare disease studies where labeled data is scarce and batches are highly imbalanced. A typical case involves patient samples collected from a few individuals across different hospitals, while healthy controls are drawn from large public datasets (Frésard *et al.* 2019). One of our recent papers (Katz *et al.* 2024) generalizes this problem setting and proposes an upper bound on the target generalization error, even when the target batch contains only few labeled samples.

In our setup, as defined in Section 3, we denote a labeled example by X=(Z,Y), where *Z* is the raw measurement (e.g. gene expression) and *Y* is the corresponding label (e.g. cell type).

As illustrated in Fig. 2, our model is a Siamese network composed of an encoder ϕ:Z→E and a classifier h:E→Y. The encoder ϕ maps raw measurements from both source and target batches into a shared embedding space E, while the classifier *h* predicts cell types based on these embeddings. The network processes samples from both batches, producing aligned embeddings and corresponding predictions.

**Figure 2:**
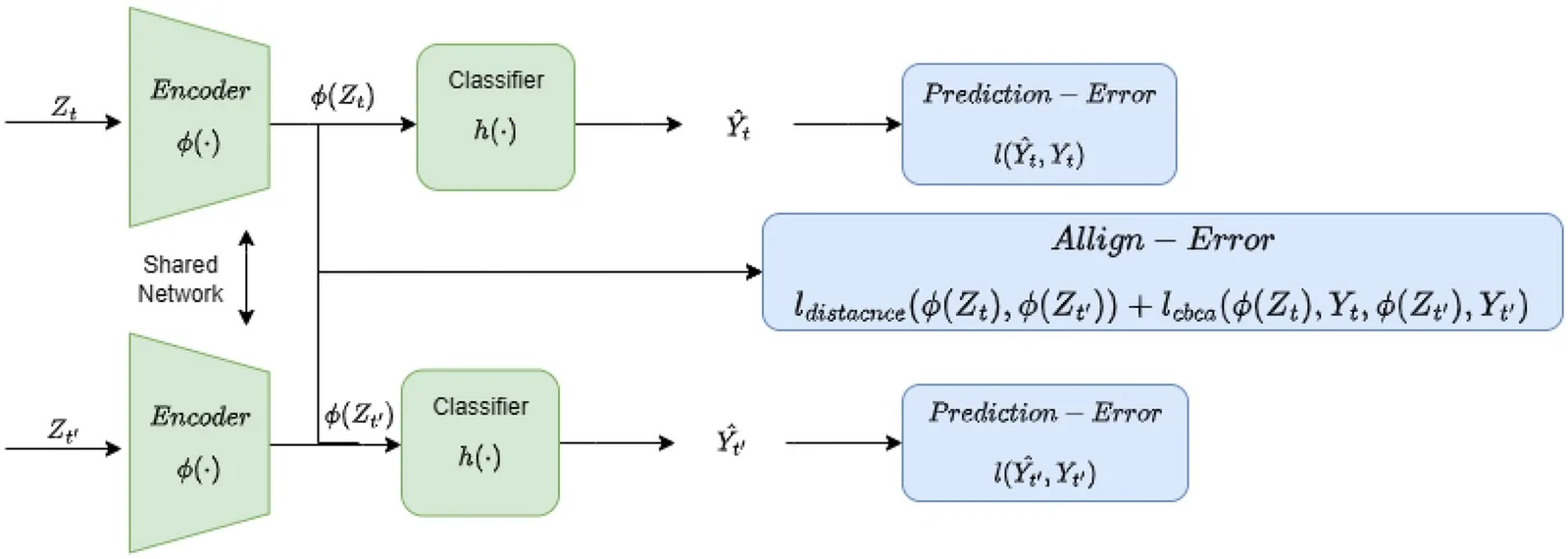
Our model, based on Siamese networks, addresses batch effect removal in supervised settings. Let $Z_{t}=\{z|(z,y)\in D^{t}\}$. $Z_{t},{\hat{Y}}_{t}$ represent the data and predicted labels from the source batch, while $Z_{t^{\prime }},{\hat{Y}}_{t^{\prime }}$ represent the data and predicted labels from the target batch. $\phi (Z_{t}),\phi (Z_{t^{\prime }})$ are the embeddings in the space map by ϕ.

It was recently shown in Katz *et al.* (2024) that the generalization error on the target batch can be upper bounded by the sum of three terms: the prediction error, the covariate shift (see Definition 5), and the cross-batch conditional agreement (CBCA; see Definition 6). For completeness, we restate the result below and provide the full derivation in the Supplementary Material, available as supplementary data at *Bioinformatics* online.

Theorem 5 (Low generalization error on future source and target samples) Let h:Z→Y be a hypothesis function, and let ℓ be a loss function satisfying the inverse triangle equality (e.g. $l_{1}$ loss)

‖ℓ(x,y)−ℓ(x,z)‖≤ℓ(y,z), ∀x,y,z.

Let $\xi_{P^{t}}(h)$ denote the expected error of h under distribution $P^{t}$. Then, for batches $t,t^{\prime }\in T$—where t represents a labeled source batch and $t^{\prime }$ a labeled target batch the following inequality holds:

$$\xi_{P^{t^{\prime }}}(h)\leq \xi_{P^{t}}(h)+Md(Q_{t},Q_{t^{\prime }})+\chi_{t,t^{\prime }}+\chi_{t^{\prime },t},$$

where

-
$\xi_{P^{t}}(h)$ and $\xi_{P^{t^{\prime }}}(h)$ represent the expected error of h on the source and target distributions, respectively (see Definition 4).
-
$d(Q_{t},Q_{t^{\prime }})$ is the total variation distance (see Definition 5).
-
$\chi_{t,t^{\prime }}$ and $\chi_{t^{\prime },t}$ measure the cross-batch conditional alignment (CBCA) error between the two distributions (see Definition 6).

Theorem 5 establishes a key theoretical result that directly supports our generalization objective in Section 3.2. It shows that the generalization error on a new (future) target batch $t^{\prime }$ can be upper bounded using only quantities computable from a source batch *t* and the relationship between the batches. Specifically, it bounds the target error $\xi_{P^{t^{\prime }}}(h)$ in terms of the source error $\xi_{P^{t}}(h)$, the marginal distributional shift $d(Q_{t},Q_{t^{\prime }})$, and the cross-batch conditional alignment terms $\chi_{t,t^{\prime }}$ and $\chi_{t^{\prime },t}$. This enables robust estimation of the target performance even in the absence of labeled target data.

In our application, we evaluate the bound on the pushforward distributions $\tilde{P^{t}}$ (see Definition 3) map by the encoder ϕ. Specifically, the quantities $\xi_{\tilde{P^{t}}}(h)$ and $\xi_{\tilde{P^{t^{\prime }}}}(h)$ correspond to the classification error of h°ϕ on the respective batches. The term $d({\tilde{Q}}_{t},{\tilde{Q}}_{t^{\prime }})$ captures the covariate shift in the embedding space, and ${\tilde{\chi }}_{tt^{\prime }}$, ${\tilde{\chi }}_{t^{\prime }t}$ are the CBCA terms on the pushforward distributions (see Definition 6).

We thus arrive at the following optimization objective:


(4) $$(\phi^{*},h^{*})=\text{arg}\underset{\left(\phi ,h\right)}{\min}\xi_{t}(h)+\xi_{t^{\prime }}(h)+Md(\phi (Z_{t}),\phi (Z_{t^{\prime }}))+{\tilde{\chi }}_{tt^{\prime }}+{\tilde{\chi }}_{t^{\prime }t},$$

Figure 3 illustrates the role of the CBCA term in capturing semantic alignment across four panels. The first panel shows the source batch, and the second displays the target batch; both are sampled from identical standard Gaussian distributions, resulting in zero distributional distance ($d(Q_{t},Q_{t^{\prime }})=0$). The third panel depics batch alignment that is based merely on marginal distributions (i.e. ignoring label information), leading to a high CBCA score. In contrast, the fourth panel presents a target batch whose cell-type labels are perfectly aligned with the source batch celltypes, resulting in a low CBCA score. These examples demonstrate that CBCA effectively detects misalignments in label configurations that are not captured by covariate alignment alone. For a detailed explanation and the complete CBCA algorithm, refer to the Supplementary Material, available as supplementary data at *Bioinformatics* online.

**Figure 3:**
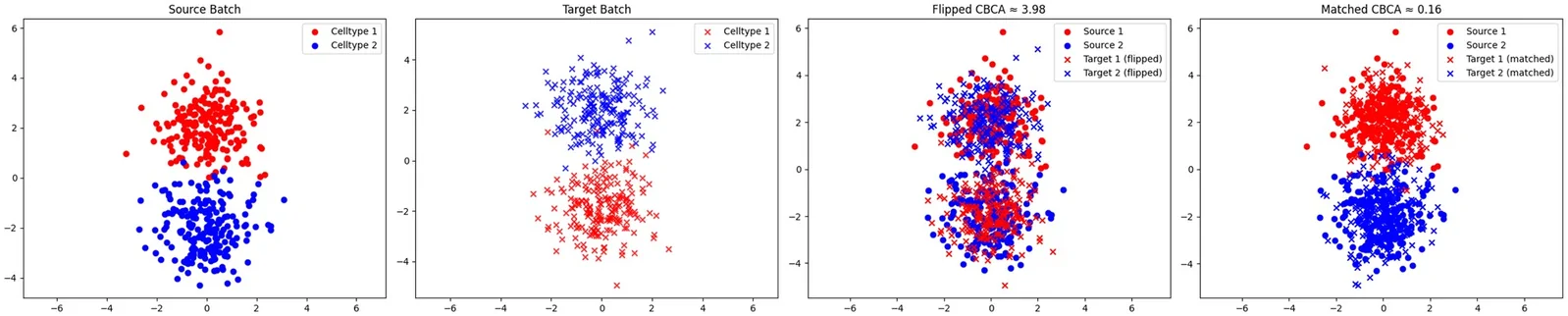
CBCA detects semantic misalignment despite identical marginal feature distributions.

## 2 Materials and methods

This section describes the experimental framework used to evaluate the proposed supervised batch effect removal (SBER) and unsupervised batch effect removal (UBER) methods. We begin by introducing the datasets and the quantitative metrics used for assessment. This is followed by a detailed analysis within the latent code space, focusing on the preservation of biological signals (Analysis), and the effectiveness of the UBER method in correcting batch effects in the original **input space** (Synthesis).

### 2.1 Metrics

To quantitatively assess model performance, we employ a suite of established metrics, each targeting a distinct aspect of the integration task. Our selection is grounded in both theoretical relevance and prior usage in recent benchmarking studies of batch correction methods (Tran *et al.* 2020, Luecken *et al.* 2022).

To evaluate the preservation of biological structure (Analysis), specifically, the separation of cell types—we use the **Adjusted Silhouette Width (ASW)**, **Adjusted Rand Index (ARI)**, and **Normalized Mutual Information (NMI)**. These metrics are standard in benchmarking studies such as Luecken *et al.* (2022), Tran *et al.* (2020):

- **ASW** quantifies the cohesion of cells within the same type and their separation from other types, adjusted to account for batch effects.
- **ARI** quantifies the similarity between predicted clusters, via *k*-means, and ground-truth labels.
- **NMI** quantifies the mutual dependence between predicted and true cell-type labels, normalized to the [0, 1] range.

To evaluate the removal of batch effects in the **input space** (Synthesis), we use the **MMD** is a non-parametric, kernel-based metric for quantifying distributional similarity between batches. Following its application in batch effect correction studies such as Shaham *et al.* (2017), we compute MMD on the top 20 principal components (PCs) of the transformed source and target batches. This choice is consistent with widely adopted benchmarking protocols, including Tran *et al.* (2020, *Genome Biology*) and the scIB framework, where distance- and neighborhood-based metrics such as kBET, ASW, and LISI are evaluated in PCA space. In this setting, MMD serves as a measure of the residual batch-related variation that remains after correction.

For a more detailed explanation of each metric, refer to the Supplementary Material, available as supplementary data at *Bioinformatics* online.

### 2.2 Baselines

To evaluate the performance of our proposed approach, we compare it against established benchmarks and commonly used batch correction methods, following prior studies Tran *et al.* (2020), Luecken *et al.* (2022). For biological conservation assessment, we adopt widely recognized tools such as harmony, liger, scanorama, scVI, and bbknn, which encompass both embedding- and correction-based strategies. The implementations of these methods follow those used in the benchmarking framework described in Luecken *et al.* (2022), ensuring consistency and reproducibility across evaluations. Additionally, we include an unintegrated baseline representing the original, uncorrected data.

To assess batch effect removal in the input space, we follow the benchmark setup of Shaham *et al.* (2017). In this setting, all compared methods—including our proposed **UBER**—operate directly on the original expression matrix rather than on a latent embedding. Specifically, we compare against:

- **Mean-variance normalization**: a straightforward normalization method aligning batch distributions by standardizing their mean and variance.
- **ComBat**: a popular empirical Bayes-based approach for batch effect adjustment.
- **PCA-based correction**: a dimensionality reduction-based strategy that removes PCs most strongly associated with batch variation.

### 2.3 Datasets

To assess the generality of our batch correction methods, we evaluate them across two broad scenarios, as illustrated in Fig. 4: (a) full-overlap, where all cell populations are shared across batches, and (b) partial-overlap, where batches contain both shared and distinct cell types. Datasets **CyTOF-P1-3M** (see Dataset 1) and **scRNA-DS2** (see Dataset 2) represent the full-overlap setting (Fig. 4a), while **scRNA-DS3** (see Dataset 3) corresponds to the partial-overlap setting (Fig. 4b), **scRNA-DS6** and **scRNA-DS7** (see Dataset 4) are included to evaluate performance in large-scale multi-batch integration settings involving multiple heterogeneous batches, diverse cell populations, and large cell counts. For clarity, we assign each dataset a unique identifier used consistently throughout this work. As illustrated in Fig. 4, datasets **CyTOF-P1-3M** (see Dataset 1) and **scRNA-DS2** (see Dataset 2) represent the full-overlap setting (Fig. 4a), while **scRNA-DS3** (see Dataset 3) corresponds to the partial-overlap setting (Fig. 4b). For clarity, we assign each dataset a unique identifier used consistently throughout this work.

**Figure 4:**
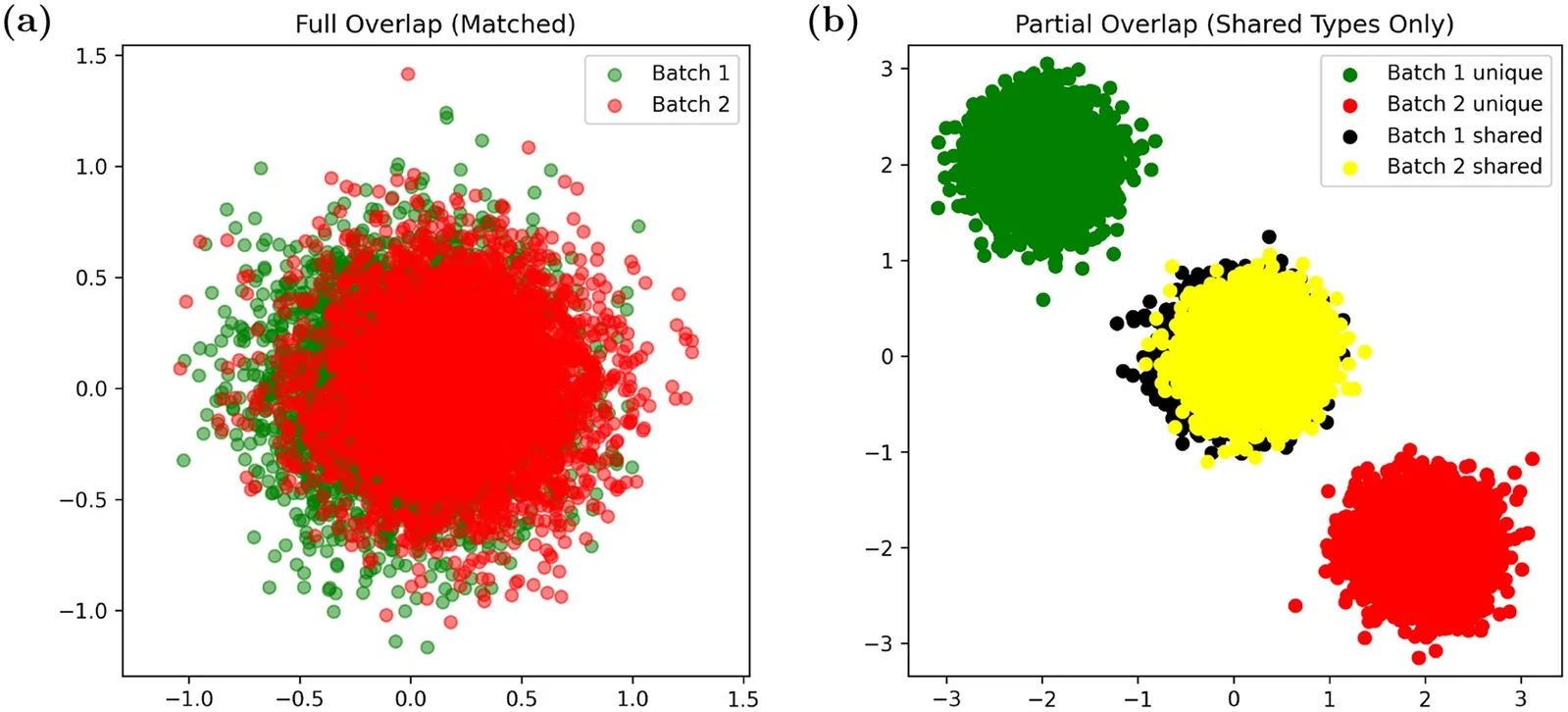
Types of batch effect removal. (a) Data after batch effect correction: when no distinct cell types are present, calibration merges the datasets into a single shared distribution. (b) Data after batch effect correction: when distinct cell types exist, calibration is applied only to the overlapping region, aligning shared cells while preserving distinct populations.

1. **CyTOF-P1-3M.** This dataset, obtained from Shaham *et al.* (2017), comprises CyTOF mass cytometry data, which measure protein levels in individual cells using antibodies conjugated to heavy metal ions. CyTOF data are known to suffer from batch effects due to instrumental variability. For evaluation, we focus on a specific test case from this dataset: the sample from **Patient 1, 90 days post-treatment** (referred to as **P1-3M**), which serves as the target in a source–target batch correction scenario. The source is the corresponding baseline sample from the same patient (day 1). Each sample is characterized by 25 protein markers, with 1800–5000 cells per sample. This setting provides a realistic and challenging benchmark for assessing batch effect correction methods.
2. **scRNA-DS2: cross-technology integration**. This dataset group evaluates robustness against batch effects introduced by different sequencing technologies, while retaining shared biological structure. It includes two complementary single-cell RNA-seq datasets with overlapping cell types but distinct technological origins:
  - **DS2a: mouse cell atlas integration**—this dataset integrates a Mouse Cell Atlas from two platforms: Microwell-Seq and Smart-Seq2. It includes 11 common cell types across nine organs, with 4239 cells and 2715 cells in each batch, respectively. The datasets share 15 006 genes. We use this dataset for **analysis**, due to its rich biological diversity and well-annotated multi-organ composition, which makes it particularly suited for evaluating biological structure preservation across batches.
  - **DS2b: hematopoietic cell integration**—this dataset combines Mouse Hematopoietic Stem and Progenitor Cells measured using SMART-seq2 and MARS-seq. It contains 1920 and 2729 cells per batch, respectively, across a shared set of 3467 highly variable genes. We use DS2b for **synthesis** because it features a well-defined batch structure and a focused cell population, providing a controlled environment for training batch correction models. Its moderate dimensionality also ensures computational efficiency and stability when working directly in the original input space. Both DS2a and DS2b assess the ability of batch correction methods to align cell populations across different sequencing technologies.
3. **scRNA-DS3: similar versus dissimilar cell types.** This dataset is constructed to evaluate the performance of batch correction methods in a nuanced scenario involving both shared and distinct cell populations. It consists of two batches:
  - **Batch 1:** *DoubleNeg, CD141, pDC*.
  - **Batch 2:** *CD1C, DoubleNeg, pDC.*
    Two cell types—*DoubleNeg and pDC*—are shared across batches, while *CD1C* and *CD141* are unique to Batch 2 and Batch 1, respectively. This setup poses a challenge for integration methods: they must align the shared cell types accurately while preserving the biological distinction between the unique cell types. The goal is to assess each method’s ability to avoid overcorrection and prevent artificial mixing of dissimilar populations.
4. **scRNA-DS6 & scRNA-DS7: large-scale multi-batch atlases.** To directly evaluate performance on complex, realistically large data ecosystems with multiple disparate sources, we appended two extended datasets from the Tran *et al.* (2020). multi-batch benchmarking suite (DS6 and DS7). These specific datasets encapsulate challenging integrations involving heterogeneous sample architectures across immense cell counts and N>2 batch conditions, providing a rigorous and demanding testbed to analyse algorithmic scalability, global multi-batch alignment precision, and rare cell-type cluster conservations. To operate in a setting of more than two batches, we select a reference batch (typically, a batch with the largest number of clusters) and correct each other batch to the reference one.

## 3 Results

### 3.1 Analysis—evaluation of biological conservation

In this section, we evaluate the effectiveness of our proposed batch correction methods, **SBER** and **UBER**, in the **code space**. The objective is to integrate data from multiple experiments while preserving true biological variation, ensuring that the corrected representation is free from batch-specific artifacts and remains suitable for downstream analyses such as cell type identification.

Table 1 presents the results, indicating that **UBER** generally surpasses current state-of-the-art methods across most datasets, including **scRNA-DS3** and **scRNA-DS2a**. For **CyTOF-P1-3M**, however, the performance is less consistent, yielding mixed outcomes. While the ASW score is high, the NMI and ARI are lower, reflecting that **UBER** does not explicitly aim to maximize cell-type clustering.

**Table 1:** Performance comparison across datasets using NMI, ARI, and ASW.^a^

	Method	NMI					ARI					ASW				
		DS3	DS2a	P1-3M	DS6	DS7	DS3	DS2a	P1-3M	DS6	DS7	DS3	DS2a	P1-3M	DS6	DS7
unsup	unintegrated	0.660	0.641	0.403	0.558	0.959	0.424	0.398	0.517	0.296	0.982	0.565	0.548	0.588	0.589	0.771
	harmony	0.713	0.666	0.381	0.520	0.957	0.628	0.465	0.488	0.250	0.981	0.586	0.625	0.550	0.579	0.778
	liger	0.702	0.690	**0.418**	–	–	0.547	0.530	**0.584**	–	–	0.539	0.510	0.539	–	–
	scanorama	**0.763**	0.730	0.345	0.561	0.959	0.749	**0.594**	0.402	0.294	0.982	0.640	0.616	0.595	0.604	0.779
	scvi	0.722	0.710	0.374	0.577	0.961	0.577	0.560	0.514	0.280	0.983	0.529	0.503	0.554	0.533	0.636
	bbknn	0.713	0.690	0.308	**0.584**	0.961	**0.758**	0.518	0.379	0.303	0.983	**0.733**	**0.626**	–	**0.614**	0.814
	SVA	0.758	0.677	0.358	0.514	0.205	0.735	0.528	0.426	**0.305**	0.116	0.633	0.607	0.587	0.545	0.540
	SAUCIE	0.694	0.662	0.343	0.020	0.380	0.709	0.484	0.433	−0.002	0.150	0.639	0.611	0.641	0.507	0.473
	UBER	0.744	**0.731**	0.233	0.517	**0.972**	0.637	0.589	0.135	0.303	**0.988**	0.582	0.586	**0.716**	0.577	**0.839**
sup (SBER)	scGen	0.831	0.732	0.156	0.420	0.587	0.859	0.442	0.034	0.174	0.291	0.662	0.635	0.582	0.820	0.615
	20%	0.851	0.871	0.661	0.692	0.982	0.894	0.880	0.821	0.453	0.993	0.854	0.826	0.948	0.866	0.869
	30%	0.816	0.891	0.740	0.736	0.026	0.847	0.903	0.869	0.563	−0.002	0.844	0.841	0.962	0.888	0.617
	40%	0.885	0.916	0.756	0.757	0.020	0.915	0.933	0.877	0.581	−0.002	0.882	0.880	0.966	0.897	0.556
	50%	0.875	0.921	0.810	0.759	0.019	0.891	0.939	0.911	0.579	0.003	0.894	0.895	0.971	0.906	0.600
	60%	0.878	0.930	0.854	**0.807**	0.987	0.908	0.947	0.935	0.732	**0.995**	0.895	0.901	0.977	**0.914**	**0.965**
	70%	**0.955**	**0.943**	**0.882**	0.805	**0.988**	**0.968**	**0.958**	**0.950**	**0.756**	**0.995**	**0.931**	**0.909**	**0.980**	0.909	0.964

a The dash (–) indicates that the metric could not be computed because of dataset input-size limitations. Bold values indicate the highest score in each dataset and metric column separately for unsupervised and supervised methods; ties are also bold.

It is important to emphasize that direct comparisons between **SBER** and unsupervised methods (e.g. Harmony, Liger, Scanorama, scVI, BBKNN, UBER) are not strictly equivalent. **SBER** leverages partial supervision, whereas the competing methods rely solely on the intrinsic structure of the data. To ensure a fair comparison, all methods were evaluated under identical preprocessing pipelines and using the same evaluation metrics.

In our experimental design, **SBER** was trained using different fractions of labeled samples from the target batch, following the practical scenario where $N_{0}\gg N_{1}$ (Section 3.2). Specifically, we evaluated **SBER** under supervision ratios ranging from 20% to 70% of labeled target data. This extended evaluation enables a systematic analysis of the robustness of the proposed framework under varying levels of annotation availability. Despite the limited supervision, **SBER** consistently achieves strong performance and outperforms all competing methods across most metrics and datasets. In general, increasing the proportion of labeled target samples leads to further improvements in alignment quality and biological conservation, demonstrating that **SBER** can effectively utilize additional supervision when available. The complete results for all supervision ratios are summarized in Table 1.

To further contextualize these quantitative results, we examine the **scRNA-DS3** dataset (see Dataset 3), specifically designed to evaluate integration of shared and batch-specific cell populations (Fig. 5). Both methods—**UBER** (panel a) and **SBER** (panel b)—effectively mix the two batches, as evidenced by the alignment of shared cell types. For **SBER**, as expected, each cell type forms distinct and well-defined clusters:

**Figure 5:**
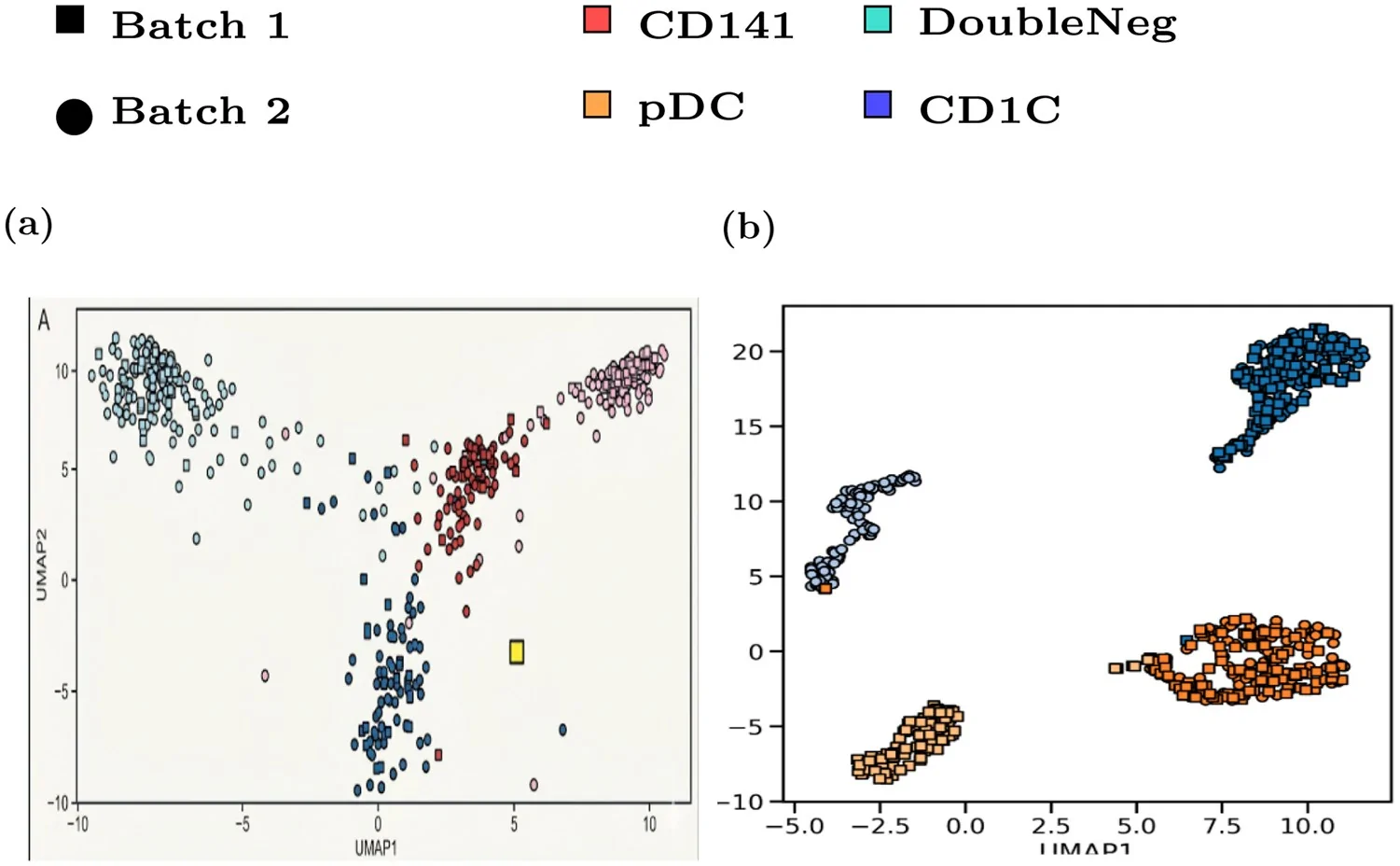
Comparison of batch correction with **UBER** (panel a) and **SBER** (panel b). Batch 1 cells are shown as squares and Batch 2 cells as circles. Both methods effectively align the two batches by integrating shared populations such as DoubleNeg and pDC. However, SBER yields more distinct and well-defined clusters, fully mixing shared cell types while preserving the separation of batch-specific populations (CD141 in Batch 1 and CD1C in Batch 2). In contrast, **UBER** shows partial overcorrection, with the shared CD141 population splitting into batch-specific subclusters.

- Shared populations such as *DoubleNeg* and *pDC* are fully integrated across batches, showing no batch-induced separation.
- Batch-specific populations (*CD141* in Batch 1 and *CD1C* in Batch 2) remain clearly separated, preserving their biological identity.

In **UBER**, integration also aligns the batches, but with a key distinction: while the *DoubleNeg* and *pDC* clusters remain well-preserved, the *CD141* population shared across batches splits into two subclusters corresponding to the individual batches. This behavior is expected, as UBER relies on reconstruction and does not explicitly aim to maximize cell-type separation.

### 3.2 Additional ablation details

For completeness, we provide extended ablation results and additional experimental configurations in the Supplementary Material (see page 6), available as supplementary data at *Bioinformatics* online. This includes a finer-grained breakdown of architectural variants, additional loss-component removals, and sensitivity analyses across different hyperparameter settings. These results further support the conclusions presented in the main text regarding the importance of adversarial alignment and CBCA regularization in improving batch correction performance.

### 3.3 Synthesis—evaluation of batch correction in input space

In this section, we evaluate our unsupervised method’s (**UBER**) ability to correct batch effects directly in the high-dimensional **input space**, a task whose difficulty varies depending on the dataset’s characteristics as illustrated in Fig. 4.

Our evaluation addresses datasets where batches contain the same underlying biological populations but differ due to technical variability, a common scenario in cross-technology or longitudinal studies. For these cases, such as the cross-technology integration benchmark (**scRNA-DS2b**, Dataset 2) and the CyTOF dataset (**CyTOF-P1-3M**, Dataset 1), UBER demonstrates strong performance. As shown in Fig. 6, UMAP visualizations (panels a and b) reveal that UBER achieves superior mixing of cells across batches compared to the uncorrected state. The quantitative assessment in panel (c) corroborates this, showing that UBER consistently achieves the lowest MMD scores among all benchmarked methods, indicating the most effective distributional alignment in the input space.

**Figure 6:**
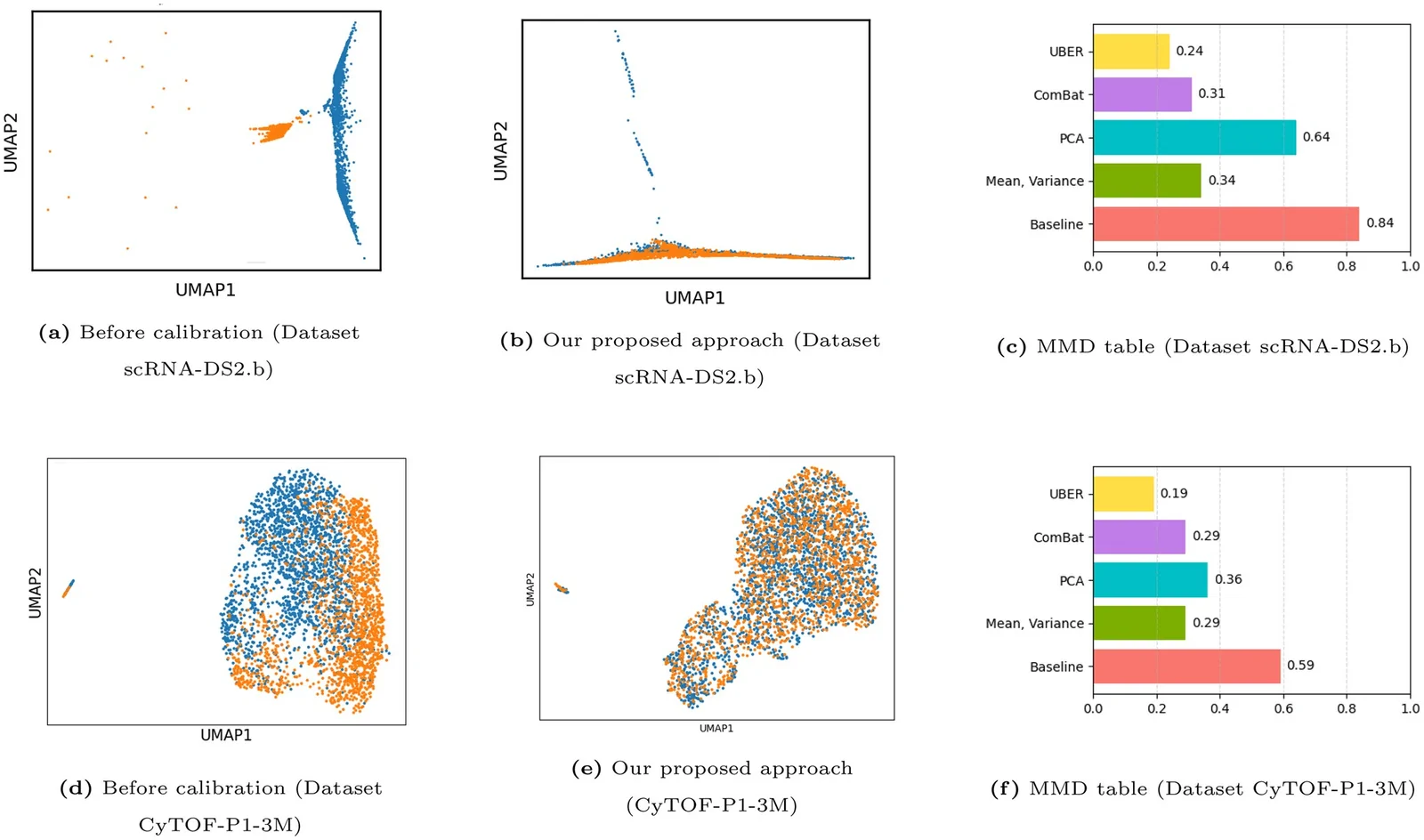
Batch correction in input space. UMAP visualizations [color represents batch in panels (a, b, d, e)] demonstrating the impact of the UBER method on batch correction across two datasets. Panels (a, d) show the unaligned data before calibration, where batch effects are evident. Panels (b, e) display the data after applying the UBER method, revealing a significant improvement in alignment between batches. Panels (c, f) illustrate the corresponding alignment scores, quantified using MMD, which indicates the extent of alignment between the calibrated data and the target batch. The figure highlights the efficacy of UBER in reducing batch effects compared to traditional methods. Lower is better.

## 4 Conclusion

We introduced two novel methods for batch effect removal: **UBER**, an unsupervised method, and **SBER**, a supervised approach. Experiments on CyTOF and scRNA-seq data show that SBER outperforms state-of-the-art methods in biological conservation and clustering, while UBER offers strong correction with some limitations in clustering accuracy.

Both methods are theoretically grounded, with formal proofs provided in the Supplementary Material, available as supplementary data at *Bioinformatics* online. Although our experiments focus on two batches, the methods naturally extend to multiple batches, increasing their applicability. This is particularly valuable in supervised settings, where prior knowledge across batches can be leveraged to guide correction.

Our framework provides robust solutions for supervised and unsupervised scenarios, contributing to more accurate and scalable data integration in bioinformatics.

## Supplementary Material

btag735_Supplementary_Data
