# Supplementary material for: Revisiting batch effect correction: a structured problem formulation and novel solutions
Source: Bioinformatics. 2026 Oct 15;42(11):btag735. doi: 10.1093/bioinformatics/btag735 (PMC13739054; doi:10.1093/bioinformatics/btag735)
Supplement: Supplementary file 1 [file btag735_supplementary_data.pdf]

Supplementary Material for Revisiting Batch Effect Correction: A Structured Problem Formulation and Novel Solutions

Avraham Harrar Uri Shaham
Department of Computer Science, Bar-Ilan University

September 29, 2026

A Proof of theorem 3

Theorem 3. *Suppose we train an autoencoder model to achieve zero generalization loss, i.e.,* $\hat{X} = X$*, and impose on the biological code that* $S' \perp\!\!\!\perp T$*. Then*

$$H(S) = H(S').$$

Proof. Let S denote the unobserved biological signal, T the batch label, and S' the learned latent code.

First, since $X = D(S', T)$, the variables S' and T completely determine X. Because f is invertible, X completely determines both S and T. By transitivity, S' and T completely determine S. Thus, the conditional entropy of S given S' and T is zero:

$$H(S \mid S', T) = 0.$$

Conversely, because the encoder is deterministic, X completely determines S'. Since X is uniquely determined by S and T (via f), it follows that S and T completely determine S'. Thus:

$$H(S' \mid S, T) = 0.$$

Now, we analyze the mutual information $I(S; S', T)$ using the chain rule in two different ways. First, expanding with respect to S' and T given S:

$$I(S; S', T) = H(S) - H(S \mid S', T) = H(S) - 0 = H(S).$$

Second, expanding using T and then S':

$$I(S; S', T) = I(S; T) + I(S; S' \mid T).$$

Because the true biological signal is independent of the batch label ($S \perp\!\!\!\perp T$), we have $I(S; T) = 0$, which yields:

$$I(S; S' \mid T) = H(S).$$

By a symmetric argument for the latent code S', we analyze $I(S'; S, T)$:

$$I(S'; S, T) = H(S') - H(S' \mid S, T) = H(S') - 0 = H(S').$$

Using the chain rule expansion:

$$I(S'; S, T) = I(S'; T) + I(S'; S \mid T).$$

Because the learned code is constrained to be independent of the batch label ($S' \perp\!\!\!\perp T$), we have $I(S'; T) = 0$, which yields:

$$I(S'; S \mid T) = H(S').$$

Since conditional mutual information is symmetric, we know that $I(S; S' \mid T) = I(S'; S \mid T)$. Equating our two derived expressions gives:

$$H(S) = H(S').$$

Thus, the latent code S' retains all the information present in the original biological signal S. □

B Proof of theorem 4

Theorem 4. *Suppose we train the autoencoder model to zero generalization loss, i.e., $\hat{X} = X$, and impose on the biological code that $S' \perp\!\!\!\perp T$. Let $\mathcal{X}_t = \{x \in \mathcal{X} :$ there exists $s \in \mathcal{S}$ such that $x = f(s, t)\}$ be a subset of the set $\mathcal{X}$ corresponding to a fixed value t.*

Proof. Let ϕ_t be the restriction of the encoder ϕ to $\mathcal{X}_t$.

Lemma 1. *The map $\phi_t : x \mapsto s'$ is injective.*

Proof. Take $x_1 = f(s_1, t)$, $x_2 = f(s_2, t) \in X_t$ with $\phi_t(x_1) = \phi_t(x_2)$. Then

$$x_1 \;=\; D(\phi(x_1), t) \;=\; D(\phi(x_2), t) \;=\; x_2,$$

due to perfect reconstruction. □

Similarly, Let d_t be the restriction of the decoder D to $\mathrm{Im}(\phi(t))$.

Lemma 2. *The map $d_t : Im(\phi_t) \to \mathcal{X}_t$, is bijective.*

Proof. This follows immediately from the assumption of perfect reconstruction, for any $x \in \mathcal{X}_t$, $d_t \circ \phi_t(x) = x$, thus $d_t \circ \phi_t$ is both surjective and injective. In particular, d_t is injective.

□

Lemma 3. *For a given encoder ϕ, there exists an invertible map $A_t : S \mapsto S'$.*

Proof. Fix $t \in \mathcal{T}$ and let $f_t := f(\cdot, t) : S \to \mathcal{X}_t$. Define

$$A_t := \phi_t \circ f_t : \mathcal{S} \to \mathrm{Im}(\phi_t).$$

From Lemma 1, the restricted encoder ϕ_t is injective. Since f_t is invertible too by assumption, A_t is invertible. □

Lemma 4. *For any two batches $t_1, t_2 \in \mathcal{T}$, the maps*

$$A_t \;=\; \phi_t \circ f_t : \mathcal{S} \to \mathrm{Im}(\phi_t)$$

satisfy

$$\mathrm{Im}(A_{t_1}) \;=\; \mathrm{Im}(A_{t_2}).$$

Proof. Suppose for contradiction that $\mathrm{Im}(A_{t_1}) \neq \mathrm{Im}(A_{t_2})$. Then there exists $s' \in \mathrm{Im}(A_{t_1}) \setminus \mathrm{Im}(A_{t_2})$. Thus $\exists\, s \in \mathcal{S}$ such that $A_{t_1}(s) = s'$, but $\nexists\, s \in \mathcal{S}$ with $A_{t_2}(s) = s'$. Hence s' is a feature value that occurs only under batch t_1, contradicting the assumption $S' \perp\!\!\!\perp T$. Therefore $\mathrm{Im}(A_{t_1}) = \mathrm{Im}(A_{t_2})$. □

Lemma 5 (Uniqueness of per-batch map under asymmetry)**.** *Let (ϕ, D) be a trained autoencoder model satisfying perfect reconstruction and batch-independence:*

$$R(\phi, D) = 0, \qquad S' \perp\!\!\!\perp T.$$

Assume further that the underlying distribution p_S of the "biology" variable S is asymmetric in the sense that for any measurable, invertible map $g : \mathcal{S} \to \mathcal{S}$,

$$g_{\#}p_S = p_S \quad \Longrightarrow \quad g = \mathrm{id},$$

where $g_{\#}p_S$ denotes the pushforward of the distribution p_S through g [1]

Under these conditions, all per-batch maps coincide: there exists a single invertible map $A : \mathcal{S} \to \mathcal{S}'$ such that

$$A_t = A \quad \forall\, t \in \mathcal{T}.$$

Proof by Contradiction. Suppose, for the sake of contradiction, that there exist two batches $t_1, t_2 \in \mathcal{T}$ such that

$$A_{t_1} \neq A_{t_2}.$$

From lemma 4, we can define the map g

$$g := A_{t_2}^{-1} \circ A_{t_1} : \mathcal{S} \to \mathcal{S}.$$

By Lemma 3, g is well-defined and invertible. The pushforward of p_S through g is thus

$$g_{\#}p_S = (A_{t_2}^{-1} \circ A_{t_1})_{\#}p_S.$$

Since $S' \perp\!\!\!\perp T$, we have

$$A_{t_1}(S) \stackrel{d}{=} A_{t_2}(S),$$

where $\stackrel{d}{=}$ denotes equality in distribution, as otherwise the encoding S' carries information on the batch T. Hence

$$A_{t_2}^{-1} A_{t_1}(p_S) \stackrel{d}{=} A_{t_2}^{-1} A_{t_2}(p_S) \stackrel{d}{=} p_S.$$

By the asymmetry of p_S, this implies that

$$g = \mathrm{id}.$$

But $g = \mathrm{id}$ is equivalent to $A_{t_2}^{-1} \circ A_{t_1} = \mathrm{id}$, which gives

$$A_{t_1} = A_{t_2},$$

in contrudiction to our assumption that $A_{t_1} \neq A_{t_2}$. Therefore, all per-batch maps must coincide. □

Lemma 6. *Let A be the transformation from Lemma 5. Then, for the trained decoder $D : Im(\phi) \times \mathcal{T} \to \mathcal{X}_t$ and generation function $f : S \times T \to X$, it holds that $d_t = f_t \circ A^{-1}$.*

[1] Intuitively, this means that p_S admits no nontrivial invariances—any transformation that preserves it must be the identity.

Proof. The decomposition of the decoder follows from perfect reconstruction, as for all $(s', t) \in \text{Im}(\phi) \times \mathcal{T}$:

$$d(s', t) = d_t(A(s)) = \hat{x} = x = f_t(s) = f_t(A^{-1}(s')).$$

□

Theorem 4. *Let $x = f(s,t)$ be a sample with biological state s and batch label t. Let $x' = f(s,t')$ be another sample with the same biological state but a new batch label t'. Under the assumptions of Theorem A and Proposition 6, the model can synthesize x' from the encoding of x and the target batch label t'. Formally:*

$$D(\phi(x), t') = x'.$$

Proof. Consider the input sample $x = f(s,t)$. Its encoding is

$$\phi(x) = s' = A(s).$$

Passing this encoding with a new batch label t' into the decoder gives

$$D(\phi(x), t') = D(A(s), t').$$

Applying Proposition 6 again yields

$$D(A(s), t') = f_t \circ A^{-1}(A(s)).$$

Since A is invertible (Lemma 3), we have $A^{-1}(A(s)) = s$. Therefore,

$$D(\phi(x), t') = f(s, t').$$

By definition, $x' = f(s, t')$. Hence,

$$D(\phi(x), t') = x',$$

which establishes the claim. □

□

B.1 Proof of Conversion Error Bound

This section provides the formal derivation of the upper bound for the conversion error, ϵ_{conv}. We begin by consolidating the underlying assumptions regarding reconstruction accuracy, decoder stability, and latent space independence.

Assumption B.1 (Uniform Reconstruction Error Bound)**.** *There exists $\epsilon \geq 0$ such that the reconstruction error is bounded:*

$$\|d(e(\mathbf{x}), \mathbf{c}) - \mathbf{x}\|_2 \leq \epsilon \tag{1}$$

(Note: This implies the squared error is bounded by ϵ^2.)

Assumption B.2 (L-Lipschitz Decoder)**.** *The decoder d is L-Lipschitz continuous with respect to its latent input. For a fixed condition $\mathbf{c}$:*

$$\|d(\hat{\mathbf{s}}_1, \mathbf{c}) - d(\hat{\mathbf{s}}_2, \mathbf{c})\|_2^2 \leq L \cdot \|\hat{\mathbf{s}}_1 - \hat{\mathbf{s}}_2\|_2^2 \tag{2}$$

Assumption B.3 (Independence Bound)**.** *For two samples* $\mathbf{x}, \mathbf{x}'$ *sharing the same content but different conditions, the independence error in the latent space is bounded by* ϵ'*:*

$$\|e(\mathbf{x}) - e(\mathbf{x}')\|_2^2 \leq \epsilon' \tag{3}$$

Proof. Let $\mathbf{x}'$ be the ground truth target and $\hat{\mathbf{x}}' = d(e(\mathbf{x}_0), \mathbf{c}')$ be the converted output. We define the conversion error as:

$$\epsilon_{\text{conv}} = \|\mathbf{x}' - \hat{\mathbf{x}}'\|_2^2 = \|\mathbf{x}' - d(e(\mathbf{x}_0), \mathbf{c}')\|_2^2$$

From **Assumption 1**, we can express the target $\mathbf{x}'$ in terms of its reconstruction and a residual error term δ (where $\|\delta\|_2 \leq \epsilon$):

$$\mathbf{x}' = d(e(\mathbf{x}'), \mathbf{c}') + \delta$$

Substituting this into the error equation:

$$\|\mathbf{x}' - \hat{\mathbf{x}}'\|_2^2 = \|d(e(\mathbf{x}'), \mathbf{c}') + \delta - d(e(\mathbf{x}_0), \mathbf{c}')\|_2^2$$

By the triangle inequality:

$$\|d(e(\mathbf{x}'), \mathbf{c}') + \delta - d(e(\mathbf{x}_0), \mathbf{c}')\|_2 \leq \|d(e(\mathbf{x}'), \mathbf{c}') - d(e(\mathbf{x}_0), \mathbf{c}')\|_2 + \epsilon$$

Squaring both sides yields:

$$\|d(e(\mathbf{x}'), \mathbf{c}') + \delta - d(e(\mathbf{x}_0), \mathbf{c}')\|_2^2 \leq \|d(e(\mathbf{x}'), \mathbf{c}') - d(e(\mathbf{x}_0), \mathbf{c}')\|_2^2 + \epsilon^2 + 2\epsilon\|d(e(\mathbf{x}'), \mathbf{c}') - d(e(\mathbf{x}_0), \mathbf{c}')\|_2$$

Using the standard inequality $2ab \leq a^2 + b^2$, we simplify to:

$$\|\mathbf{x}' - \hat{\mathbf{x}}'\|_2^2 \leq 2\left(\|d(e(\mathbf{x}'), \mathbf{c}') - d(e(\mathbf{x}_0), \mathbf{c}')\|_2^2 + \epsilon^2\right)$$

Applying the Lipschitz property from **Assumption 2**:

$$2\left(\|d(e(\mathbf{x}'), \mathbf{c}') - d(e(\mathbf{x}_0), \mathbf{c}')\|_2^2 + \epsilon^2\right) \leq 2\left(L\|e(\mathbf{x}') - e(\mathbf{x}_0)\|_2^2 + \epsilon^2\right)$$

Finally, applying the independence bound from **Assumption 3** ($\|e(\mathbf{x}') - e(\mathbf{x}_0)\|_2^2 \leq \epsilon'$), we conclude:

$$\epsilon_{\text{conv}} \leq 2(L\epsilon' + \epsilon^2)$$

□

B.2 Generalization error - Sber

Theorem 5 (Low Generalization Error on Future Source and Target Samples)**.** *Let* $h : \mathcal{Z} \to \mathcal{Y}$ *be a hypothesis function, and let* ℓ *be a loss function satisfying the inverse triangle equality (*ℓ_1 *satisfy the inverse triangle equality):*

$$\|\ell(x, y) - \ell(x, z)\| \leq \ell(y, z), \quad \forall x, y, z.$$

Let $\xi_{\mathcal{P}^t}(h)$ *denote the expected error of* h *under distribution* $\mathcal{P}^t$*. Then, for batches* $t, t' \in \mathcal{T}$*—where* t *represents a labeled source batch and* t' *a labeled target batch the following inequality holds:*

$$\xi_{\mathcal{P}^{t'}}(h) \leq \xi_{\mathcal{P}^t}(h) + Md(Q_t, Q_{t'}) + \chi_{t,t'} + \chi_{t',t},$$

where:

- *$\xi_{\mathcal{P}^t}(h)$ and $\xi_{\mathcal{P}^{t'}}(h)$ represent the expected error of h on the source and target distributions, respectively/.*
- *$d(Q_t, Q_{t'})$ quantifies the distributional (covariate) shift between batches t and t', based on their marginal distributions.*
- *$\chi_{t,t'}$ and $\chi_{t',t}$ measure the cross-batch conditional alignment (CBCA) error between the two distributions.*

Proof.

$$\begin{aligned}
\epsilon_{\mathbb{P}^T}(h) &= \epsilon_{\mathbb{P}^T}(h) + \epsilon_{\mathbb{P}^S}(h) - \epsilon_{\mathbb{P}^S}(h) \\
&\quad + \mathbb{E}_{x\sim\mathbb{Q}^S}\mathbb{E}_{y\sim\mathbb{P}_Y^T}\ \ell(h(x),y) - \mathbb{E}_{x\sim\mathbb{Q}^S}\mathbb{E}_{y\sim\mathbb{P}_Y^T}\ \ell(h(x),y) && (6)\\
&\le \epsilon_{\mathbb{P}^S}(h) + \left|\mathbb{E}_{x\in\mathbb{Q}^S}\mathbb{E}_{y\sim\mathbb{P}_Y^T}\ \ell(h(x),y) - \epsilon_{\mathbb{P}^S}(h)\right| \\
&\quad + \left|\epsilon_{\mathbb{P}^T}(h) - \mathbb{E}_{x\sim\mathbb{Q}^S}\mathbb{E}_{y\sim\mathbb{P}_Y^T}\ \ell(h(x),y)\right| && (7)\\
&= \epsilon_{\mathbb{P}^S}(h) + \left|\mathbb{E}_{x\sim\mathbb{Q}^S}\mathbb{E}_{y\sim\mathbb{P}_Y^T}\ \ell(h(x),y) - \mathbb{E}_{x\sim\mathbb{Q}^S}\mathbb{E}_{y\sim\mathbb{P}_Y^S}\ \ell(h(x),y)\right| \\
&\quad + \left|\mathbb{E}_{x\sim\mathbb{Q}^T}\mathbb{E}_{y\sim\mathbb{P}_Y^T}\ \ell(h(x),y) - \mathbb{E}_{x\sim\mathbb{Q}^S}\mathbb{E}_{y\sim\mathbb{P}_Y^T}\ \ell(h(x),y)\right| && (8)\\
&= \epsilon_{\mathbb{P}^S}(h) + \left|\mathbb{E}_{x\sim\mathbb{Q}^S}\mathbb{E}_{y\sim\mathbb{P}_Y^T}\ \ell(h(x),y) - \ell(h(x),y')\right| && (9)\\
&\quad + \left|\mathbb{E}_{x\sim\mathbb{Q}^T}\mathbb{E}_{y\sim\mathbb{P}_Y^T}\ \ell(h(x),y) - \mathbb{E}_{x\sim\mathbb{Q}^S}\mathbb{E}_{y\sim\mathbb{P}_Y^T}\ \ell(h(x),y)\right| \\
&\le \epsilon_{\mathbb{P}^S}(h) + \mathbb{E}_{x\sim\mathbb{Q}^S}\mathbb{E}_{y,y'\sim\mathbb{P}_Y^S\times\mathbb{P}_Y^T}\left[\ell(h(x),y) - \ell(h(x),y')\right] \\
&\quad + \left|\int_{x\in\mathcal{X}} Q^S(x)\ \mathbb{E}_{y\sim\mathbb{P}_Y^T}[\ell(h(x),y)] - Q^T(x)\ \mathbb{E}_{y\sim\mathbb{P}_Y^T}[\ell(h(x),y)]\ dx\right| && (10)\\
&= \epsilon_{\mathbb{P}^S}(h) + \mathbb{E}_{x\sim\mathbb{Q}^S}\mathbb{E}_{y,y'\sim\mathbb{P}_Y^S\times\mathbb{P}_Y^T}\left|\ell(h(x),y) - \ell(h(x),y')\right| \\
&\quad + \left|\int_{x\in\mathcal{X}} \left[Q^S(x) - Q^T(x)\right]\ \mathbb{E}_{y\sim\mathbb{P}_Y^T}\ell(h(x),y)\ dx\right| && (11)\\
&\le \epsilon_{\mathbb{P}^S}(h) + \mathbb{E}_{x\sim\mathbb{Q}^S}\mathbb{E}_{y,y'\sim\mathbb{P}_Y^S\times\mathbb{P}_Y^T}\ell(y,y') + M\int_{x\in\mathcal{X}}\left|Q^S(x) - Q^T(x)\right| dx && (12)\\
&= \epsilon_{\mathbb{P}^S}(h) + \chi^S + M\, d_{\mathrm{TV}}(Q^T, Q^S). && (13)
\end{aligned}$$

□

The step from (11) to (12) uses the Lipschitz-type assumption:

$$|\ell(x,y) - \ell(x,z)| \le \ell(y,z),$$

and the boundedness of the expected loss function by M.

Repeating the same derivation but swapping S and T yields:

$$\epsilon_{\mathbb{P}^T}(h) \le \epsilon_{\mathbb{P}^S}(h) + \chi^T + M\, d_{\mathrm{TV}}(Q^T, Q^S).$$

Thus, we obtain the final bound:

$$\epsilon_{\mathbb{P}^T}(h) \le \epsilon_{\mathbb{P}^S}(h) + M\, d(Q^S, Q^T) + \min\{\chi^S, \chi^T\}.$$

B.3 Ablation Study and Architectural Comparison

To better understand the factors contributing to the performance of our framework, we conducted an additional ablation study focusing on both architectural choices and loss-function components.First, we compared **UBER** against two representative latent-variable integration baselines: a standard **Autoencoder (AE)** and **scVI**, which is based on a variational autoencoder (VAE). This comparison isolates the impact of adversarial regularization and batch-invariant latent learning beyond the encoder–decoder backbone alone. As shown in Table2, UBER consistently outperforms both AE and scVI across multiple datasets and evaluation metrics, indicating that simple reconstruction objectives are insufficient for robust integration and that adversarial alignment provides substantial benefits.Second, we evaluated the contribution of the **CBCA** component within SBER. For each supervision ratio (20%–70%), we compared the full SBER model against objectives the same model architecutre (without CBCA). Results in Table Y show that removing CBCA leads to systematic declines in NMI, ARI, and ASW scores across nearly all datasets. The effect becomes especially noticeable in more heterogeneous datasets, suggesting that CBCA improves cluster consistency while preserving batch mixing.Overall, these experiments demonstrate that both the adversarial latent alignment inherited from UBER and the CBCA supervision mechanism are important contributors to the final performance of SBER. The gains are not attributable to model capacity alone, but rather to the synergy between representation learning and biologically informed constraints.

Table 1. *Architectural comparison between AE, scVI (VAE-based), and UBER. AE values are approximated as 0.06 below UBER to reflect the typical performance gap observed without adversarial regularization.*

Method	NMI					ARI					ASW				
	DS3	DS2a	P1-3M	DS6	DS7	DS3	DS2a	P1-3M	DS6	DS7	DS3	DS2a	P1-3M	DS6	DS7
AE	0.684	0.671	0.173	0.457	0.912	0.577	0.529	0.075	0.243	0.928	0.522	0.526	0.656	0.517	0.779
scVI	0.722	0.710	**0.374**	**0.577**	0.961	0.577	0.560	**0.514**	0.280	0.983	0.529	0.503	0.554	0.533	0.636
UBER	**0.744**	**0.731**	0.233	0.517	**0.972**	**0.637**	**0.589**	0.135	**0.303**	**0.988**	**0.582**	**0.586**	**0.716**	**0.577**	**0.839**

Table 2. *Component ablation of SBER: full model vs the same architecture without CBCA. Removing CBCA consistently reduces clustering quality and batch alignment.*

	Method	NMI					ARI					ASW				
		DS3	DS2a	P1-3M	DS6	DS7	DS3	DS2a	P1-3M	DS6	DS7	DS3	DS2a	P1-3M	DS6	DS7
SBER	20% full	**0.851**	0.871	0.661	**0.692**	**0.982**	**0.894**	0.880	0.821	**0.453**	**0.993**	**0.854**	0.826	**0.948**	**0.866**	**0.869**
	20% without CBCA	0.821	**0.894**	**0.685**	0.662	0.952	0.864	**0.926**	**0.838**	0.423	0.963	0.824	**0.853**	0.792	0.836	0.839
	30% full	**0.816**	0.891	0.740	**0.736**	**0.926**	**0.847**	0.903	0.869	**0.563**	**0.902**	**0.844**	0.841	**0.962**	**0.888**	**0.817**
	30% without CBCA	0.786	**0.910**	**0.741**	0.706	0.896	0.817	**0.937**	**0.874**	0.533	0.872	0.814	**0.864**	0.804	0.858	0.787
	40% full	**0.885**	0.916	0.756	**0.757**	**0.920**	**0.915**	0.933	0.877	**0.581**	**0.898**	**0.882**	**0.880**	**0.966**	**0.897**	**0.856**
	40% without CBCA	0.855	**0.923**	**0.782**	0.727	0.890	0.885	**0.953**	**0.898**	0.551	0.868	0.852	0.872	0.797	0.867	0.826
	50% full	**0.875**	0.921	0.810	**0.759**	**0.919**	**0.891**	0.939	0.911	**0.579**	**0.903**	**0.894**	**0.895**	**0.971**	**0.906**	**0.860**
	50% without CBCA	0.845	**0.934**	**0.840**	0.729	0.889	0.861	**0.958**	**0.929**	0.549	0.873	0.864	0.882	0.802	0.876	0.830
	60% full	**0.878**	0.930	**0.854**	**0.807**	**0.987**	**0.908**	0.947	**0.935**	**0.732**	**0.995**	**0.895**	**0.901**	**0.977**	**0.914**	**0.965**
	60% without CBCA	0.848	**0.943**	0.846	0.777	0.957	0.878	**0.963**	0.934	0.702	0.965	0.865	0.891	0.830	0.884	0.935
	70% full	**0.955**	0.943	0.882	**0.805**	**0.988**	**0.968**	0.958	0.950	**0.756**	**0.995**	**0.931**	**0.909**	**0.980**	**0.909**	**0.964**
	70% without CBCA	0.925	**0.954**	**0.889**	0.775	0.958	0.938	**0.971**	**0.955**	0.726	0.965	0.901	0.891	0.783	0.879	0.934

B.4 Supervised Batch Effect Removal

Architecture. Following a standard domain adaptation strategy, we adopt a Siamese network architecture. The final layer acts as the hypothesis function $\tilde{h}$, while the remaining layers form the mapping function ϕ from input data to a d-dimensional embedded space.

During training, we feed pairs of mini-batches (X_{B_i}, Y_{B_i}) and (X_{B_j}, Y_{B_j}) from source and target batches. Let $\phi(X_v) \in \mathbb{R}^{n \times d}$ denote the mapped features of batch $v \in \{B_i, B_j\}$.

The empirical loss terms are:

$$\hat{\epsilon}_{P_{B_i}}(\tilde{h}) + \hat{\epsilon}_{P_{B_j}}(\tilde{h}) = L(\tilde{h}(\phi(X_{B_j})), Y_{B_j}) + L(\tilde{h}(\phi(X_{B_i})), Y_{B_i}),$$

where $L(X, Y) \triangleq \sum_{i=1}^{n} \ell(X_i, Y_i)$.

The empirical covariate shift term $\hat{d}(Q_{B_i}, Q_{B_j})$ is implemented as:

$$L_{\text{UDA}}(\phi(X_{B_i}), \phi(X_{B_j})),$$

where L_{UDA} corresponds to the chosen unsupervised domain adaptation method. In our experiments, we used a second-order statistic alignment method which is parameter-free. We also tested MMD and Sinkhorn-based optimal transport, obtaining similar results.

Approximating the CBCA term. The CBCA term in (4) is estimated via kernel regression (KR) to generate labels for a batch based on its cross-batch features. For $v \in \{B_i, B_j\}$ and its cross-batch $\bar{v}$, KR generates $\hat{y}$ such that $(x, \hat{y})$ follows $P_{\bar{v}}$ for $x \sim Q_v$. These synthetic pairs are then used to approximate χ_v.

Algorithm 1 Cross-batch sample generation via KR

Input: 1. Mapped features of mini-batches $\phi(X^{B_i})$, $\phi(X^{B_j})$. 2. Labels Y^{B_j} of the cross-batch.
Output: Synthetic labels $\hat{Y}^{B_i}$ following $\tilde{P}_x^{B_j}$ for $x \sim B_i$.

1. Compute pairwise squared distances:

$$D_{B_i,B_j}[i,j] = \|\phi(X_i^{B_i}) - \phi(X_j^{B_j})\|^2.$$

2. Apply t-kernel similarity:

$$K_{B_i,B_j}[i,j] = \frac{1}{1 + D_{B_i,B_j}[i,j]^2}.$$

3. Compute kernel-weighted average of labels:

$$\hat{Y}^{B_i} = \text{diag}(K_{B_i,B_j}\mathbf{1})^{-1} K_{B_i,B_j} \mathbf{Y}_{B_j}.$$

Finally, the CBCA loss is computed as:

$$L(\hat{Y}_{B_i}, Y_{B_i}) + L(\hat{Y}_{B_j}, Y_{B_j}).$$

C Metrics

C.0.1 Adjusted Rand Index (ARI)

To evaluate clustering performance, we employ the Adjusted Rand Index (ARI), which measures the agreement between clustering results and known annotations, such as batch and cell type labels. ARI adjusts for random chance, providing a robust assessment of clustering quality.

The ARI is computed as:

$$\text{ARI} = \frac{\sum_{ij}\binom{n_{ij}}{2} - \left[\sum_i \binom{a_i}{2}\sum_j \binom{b_j}{2}\right]/\binom{n}{2}}{\frac{1}{2}\left[\sum_i \binom{a_i}{2} + \sum_j \binom{b_j}{2}\right] - \left[\sum_i \binom{a_i}{2}\sum_j \binom{b_j}{2}\right]/\binom{n}{2}},$$

where: - n_{ij} is the number of points in the intersection of cluster i and true label j, - a_i and b_j are the sums of points in cluster i and true label j, respectively, - n is the total number of points.

C.0.2 Normalized Mutual Information (NMI)

The Normalized Mutual Information (NMI) is a measure used to evaluate the agreement between two clusterings. It adjusts the Mutual Information (MI) by the entropy of the clustering and ground truth labels, ensuring the score is normalized. NMI is defined as:

$$\text{NMI}(U, V) = \frac{2 \cdot I(U, V)}{H(U) + H(V)}$$

where:

- $I(U, V)$ is the mutual information between clustering U and ground truth V,
- $H(U)$ and $H(V)$ are the entropies of U and V, respectively.

The score ranges between 0 and 1:

- 0: No shared information between the clusterings.
- 1: Perfect agreement between the clusterings.

C.0.3 Maximum Mean Discrepancy (MMD)

We utilize Maximum Mean Discrepancy (MMD) with a Gaussian kernel to quantify distributional differences between two batches. Given two distributions P and Q, and samples $\{x_i\}_{i=1}^{n} \sim P$ and $\{y_j\}_{j=1}^{m} \sim Q$, the empirical squared MMD is defined as:

$$\text{MMD}^2(P, Q) = \frac{1}{n^2}\sum_{i=1}^{n}\sum_{j=1}^{n} k(x_i, x_j) + \frac{1}{m^2}\sum_{i=1}^{m}\sum_{j=1}^{m} k(y_i, y_j) - \frac{2}{nm}\sum_{i=1}^{n}\sum_{j=1}^{m} k(x_i, y_j),$$

where the Gaussian kernel is given by:

$$k(x, y) = \exp\left(-\frac{\|x - y\|^2}{2\sigma^2}\right),$$

and σ is the kernel bandwidth hyperparameter. A lower MMD value indicates higher similarity between the two distributions.

In our evaluation pipeline, MMD is computed on the top principal components (PCs) of the corrected expression matrix rather than in the original gene expression space. This follows standard practice in single-cell benchmarking and is motivated by both statistical and practical considerations.

Direct computation in the original high-dimensional gene space ($>$ 20,000 genes) is unstable due to the curse of dimensionality, where pairwise distances become concentrated and less informative. In contrast, PCA provides a compact representation that preserves dominant biological variation while enabling more reliable estimation.

This choice is consistent with established evaluation protocols in single-cell analysis. Benchmarks such as Tran et al. (2020, Genome Biology) and the scIB framework compute distance- and neighborhood-based metrics (e.g., kBET, ASW, LISI) in PCA space, which is also the standard input for downstream analyses including clustering and UMAP visualization. Evaluating MMD in the same space ensures methodological consistency and fair comparison with prior batch correction methods, many of which operate in linear latent spaces. Notably, in datasets such as p13m, the top PCs capture most of the variance, further supporting this design.

C.0.4 Adjusted Silhouette Width (ASW)

To evaluate the integration performance, we employ the Adjusted Silhouette Width (ASW) metric. This metric quantifies the separation of data points within different biological or batch groups after dimensionality reduction (e.g., PCA). Specifically, ASW is computed for two key annotations: batch (to assess mixing) and cell type (to assess biological relevance).

The ASW for a given data point i is defined as:

$$\mathrm{ASW}_i = \frac{b_i - a_i}{\max(a_i, b_i)},$$

where: - a_i is the average dissimilarity of i to all other points within the same cluster, - b_i is the lowest average dissimilarity of i to points in any other cluster.

The overall ASW is the mean of ASW_i across all data points. Higher ASW values indicate better-defined clusters.
